# Bison body size and climate change

**DOI:** 10.1002/ece3.4019

**Published:** 2018-04-10

**Authors:** Jeff M. Martin, Jim I. Mead, Perry S. Barboza

**Affiliations:** ^1^ Department of Wildlife and Fisheries Sciences Texas A&M University College Station TX USA; ^2^ Don Sundquist Center of Excellence in Paleontology Johnson City TN USA; ^3^ The Mammoth Site Hot Springs SD USA; ^4^ Desert Laboratory on Tumamoc Hill University of Arizona Tucson AZ USA

**Keywords:** Bergmann’s rule, body size change, climate change, fossil, North America, ungulate

## Abstract

The relationship between body size and temperature of mammals is poorly resolved, especially for large keystone species such as bison (*Bison bison*). *Bison* are well represented in the fossil record across North America, which provides an opportunity to relate body size to climate within a species. We measured the length of a leg bone (calcaneal tuber, DstL) in 849 specimens from 60 localities that were dated by stratigraphy and ^14^C decay. We estimated body mass (*M*) as *M* = (DstL/11.49)^3^. Average annual temperature was estimated from δ^18^O values in the ice cores from Greenland. Calcaneal tuber length of *Bison* declined over the last 40,000 years, that is, average body mass was 37% larger (910 ± 50 kg) than today (665 ± 21 kg). Average annual temperature has warmed by 6°C since the Last Glacial Maximum (~24–18 kya) and is predicted to further increase by 4°C by the end of the 21st century. If body size continues to linearly respond to global temperature, *Bison* body mass will likely decline by an additional 46%, to 357 ± 54 kg, with an increase of 4°C globally. The rate of mass loss is 41 ± 10 kg per°C increase in global temperature. Changes in body size of *Bison* may be a result of migration, disease, or human harvest but those effects are likely to be local and short‐term and not likely to persist over the long time scale of the fossil record. The strong correspondence between body size of bison and air temperature is more likely the result of persistent effects on the ability to grow and the consequences of sustaining a large body mass in a warming environment. Continuing rises in global temperature will likely depress body sizes of bison, and perhaps other large grazers, without human intervention.

## 1 INTRODUCTION

Variation in body size of American bison (Artiodactyla, Bovidae) has been a contentious topic for more than 7 decades (Dary, 1974; Hill, Hill, & Widga, 2008; McDonald, 1981; Skinner & Kaisen, 1947). In North America, Skinner and Kaisen (1947) synthesized and synonymized 52 species of bison down to eight species using primarily skulls and horn cores which respond plastically to sexual selection. McDonald (1981) and Pinsof (1991) synthesized and synonymized those eight species of bison to five, again based on cranial morphology. *Bison priscus* and *B. latifrons,* which denote sister taxa groups to the extant bison clade and represent the larger, more giant end of the body size spectrum, appear to go extinct circa 30kya. The extant bison clade in North America traditionally includes *Bison bison*,* B. occidentalis*, and *B. antiquus*, which represent a smaller body size in comparison with the larger, giant bison (*B. priscus* and *B. latifrons*). Yet, the skulls of these smaller species still represent plastic variation, likely due to sexual selection, not representative of overall body size. To avoid the issues surrounding problems with cranial morphology, our study here focuses on the postcranial body size reconstruction, particularly of a mechanistic element to the structure of the skeleton. Our assessment is that the extant bison clade species may represent a linear chronospecies and is supported by recent ancient DNA assessments (Froese et al., 2017; Shapiro et al., 2004). Likely, *B. antiquus* and *B. occidentalis* did not go extinct, but through phenotypic and morphologic adaptation to changing climatic conditions, evolved into what is traditionally referred to as *B. bison* that we have throughout the Holocene and this is what we present below.

Extant *Bison* are one of eight ungulate genera to survive the most recent deglaciation in North America (Koch & Barnosky, 2006; Kurtén & Anderson, 1980; McDonald, 1981). *Bison bison* (the extant species in North America) has also survived a more recent near‐extinction event by market hunters in the late 19th century (Dary, 1974; McDonald, 1981). Modern bison of the early 20th‐century bottleneck have rebounded in population to approximately 400,000 bison today because of conservation efforts from public and private sectors (Gates, Freese, Gogan, & Kotzman, 2010; United States Department of Agriculture, 2016). During the Holocene in North America, *Bison* had the largest distribution of any contemporary ungulate; from Pacific to Atlantic coasts and from arctic to the tropical ecoregions (Feranec, Hadly, & Paytan, 2009; McDonald, 1981; Skinner & Kaisen, 1947). Although it is often assumed that *Bison* are obligate grazers (occasionally referred to as hyper‐grazers (MacFadden & Cerling, 1996; Leng, 2006)), *Bison* have shown to be adaptable and variable in diet selection (Bergman, Fryxell, Gates, & Fortin, 2001; Feranec & MacFadden, 2000; Miquelle, 1985; Widga, 2006). *Bison* have inhabited North America (south of 55°N latitude) for approximately 200,000 years (Barnosky et al., 2014; Bell et al., 2004; Pinsof, 1991) and have occupied Beringia for nearly 300,000 years (Froese et al., 2017; McDonald, 1981; Shapiro et al., 2004).

Despite conservation efforts, modern bison face increasing temperatures and increasing variability in climate (IPCC Working Group 1, 2014). Global temperature in the 21st century is expected to rise between 1 and 4°C above the 20th‐century average (IPCC Working Group 1, 2014). Past global and regional climates can be reconstructed using isotopic markers from ice cores and marine sediments and using limnological data such as species of pollen and diatoms, and charcoal in geological context. Currently, the longest and highest resolution records for reconstructing past atmospheric conditions are stable isotopes of oxygen (^18^O) from continental ice sheets in Greenland (<120,000 years (Alley et al., 1993)) and Antarctica (<800,000 years (Jouzel et al., 2007)). Values for δ^18^O from the Greenland Ice Sheet Project (GISP2) index decadal temperatures that would have been experienced by *Bison* in the Northern Hemisphere.

**Figure 1:**
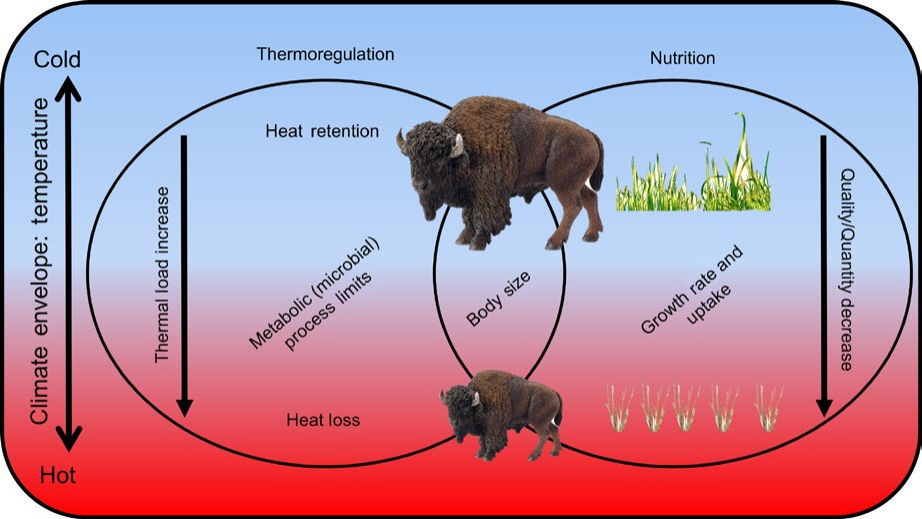
Conceptual model of the direct and indirect effects of elevated ambient temperature on body size of *Bison*

Species that are affected by climate change may alter their distribution and adapt through changes in morphology, physiology, behavior, and life history (Smith, Murray, Harding, Lease, & Martin, 2014; Smith et al., 2010). Small mammals appear to be able to adapt morphology and life history to environmental shifts within one to three generations (Crews & Gore, 2012; Mifsud et al., 2011). However, the adaptive responses of large mammals to climate change are poorly understood. In comparison with small mammals, large species can better avoid harsh environments by moving long distances, tolerate austere conditions with large bodies, and recover over multiple seasons to reproduce over long lifespans (Barboza, Parker, & Hume, 2009). Impacts of climate change on animals are twofold: direct effects of temperature on the animal (i.e., energetic load as heat) and indirect effects of temperature on the animal's food supply (Figure 1). Warm temperatures advance the seasonal growth of grasses to reduce the availability of nitrogen for growth of cattle and bison (Craine, 2013; Craine, Elmore, Olson, & Tolleson, 2010; Craine, Towne, Joern, & Hamilton, 2009; Craine et al., 2012). Ambient air temperature directly affects the costs of thermoregulation of the animal in cold winters and the ability to lose excess heat in warm summers (Long et al., 2014; Speakman & Król, 2010). Seasonal patterns of air temperature affect the onset, duration, and intensity of plant production that sets the quantity and quality of food for growth and reproduction of herbivores from spring through autumn (Albon et al., 2017; Huston & Wolverton, 2011).

At least four biological concepts attempt to explain the phenomenon of changing body size. Cope's rule recognizes the tendency of vertebrate animals to increase body size over geological time scales (Stanley, 1973). Bergmann's rule emphasizes the positive relationship between body size and latitude, which suggests that the ability to retain body heat favors larger bodies at cooler temperatures as long as net primary production is adequate for animals of large size (Bergmann, 1847; Huston & Wolverton, 2011; Watt, Mitchell, & Salewski, 2010). The Metabolic Theory of Ecology emphasizes the allometric scaling of body size and the underlying relationships between the volume of animals and the surfaces that are exposed to the environment (Brown & Sibly, 2006). The Heat Dissipation Limit Theory emphasizes heat load as a driver of body size because metabolism can produce excess energy (heat), which may be more difficult to dissipate as body size and metabolic work increase (Speakman & Król, 2010). However, these relationships alone are not sufficient to accurately project the effect of climate change on the body size of large species. Although the fossil record provides abundant evidence of changes in the body size of vertebrate animals (i.e., dinosaurs, proboscideans) that have been linked to global shifts in climate (Sander et al., 2011), taxa differ in the direction, rate, and extent of response to warming and cooling (Lovegrove & Mowoe, 2013). Among large mammals, changes in body size at a continental scale declined quickly with rising temperature but rose more slowly with cooling over the past 100 million years (Evans et al., 2012).

The relationship between body size and temperature of mammals is poorly resolved especially for ecological keystone species of large mammals, such as bison (Knapp et al., 1999). *Bison* modify ecosystems through selective grazing (Coppedge & Shaw, 1998; Fahnestock & Knapp, 1994), wallowing (Coppedge, Fuhlendorf, Engle, Carter, & Shaw, 1999; Polley & Collins, 1984), transporting nutrients (Plumb & Dodd, 1994; Towne, 2000), herd movements (Bergman et al., 2001; Van Vuren, 2001), and physical disturbance of soil and vegetation (Allred, Fuhlendorf, & Hamilton, 2011; Coppedge & Shaw, 2000). Fossilized skeletal elements can be used to study body size over long‐time frames. Our study focuses on the calcaneum (the heel bone; Figure 3), an anatomically functional element, that is, conserved evolutionarily. We used the calcaneum to estimate body mass, whereas previous authors have focused on skull metrics (McDonald, 1981; Skinner & Kaisen, 1947) that are more susceptible to sexual selection and vary widely among species. In contrast, sexual dimorphism in bison, while noticeable in modern contexts, is lost in the fossil record without adequate comparison of other representatives of the correct species at that time. Moreover, using osteometrics and ratios on postcranial elements are unable to determine the intermediate‐sized individuals within a fossil population, stated another way, mature females and immature bulls overlap in size and all immature individuals overlap in size (Lewis, Johnson, Buchanan, & Churchill, 2007). *Bison* are well represented in the fossil record across North America, which provides an opportunity to relate body size to climate within a taxon over the last 40,000 years. In this study, we used the historical and prehistorical records of *Bison* to test the hypothesis that large‐scale changes in climate drive changes in body size.

## 2 MATERIALS AND METHODS

**Figure 2:**
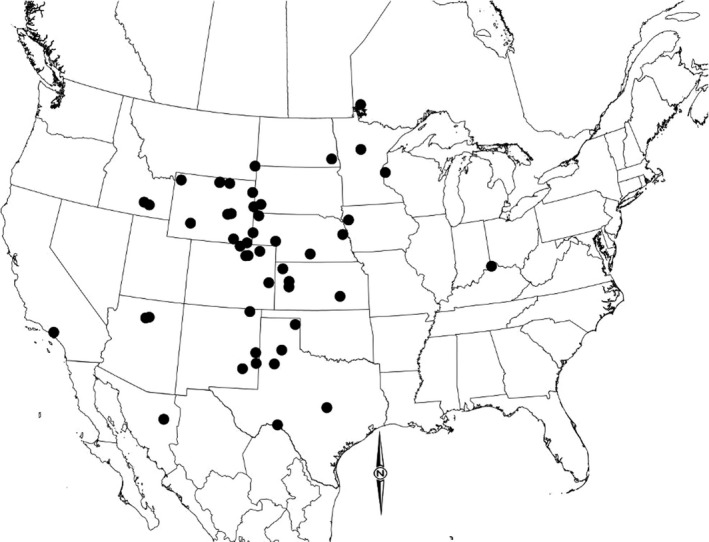
Localities (*n *=* *60) of fossil specimens in North America that correspond with body mass estimates of bison with calibrated age. Sites are further described in Data S2

We used curated specimens from modern and fossil *Bison*. Data S1 lists specimen numbers and sponsoring collections. Physiographic and chronological information about localities is summarized in Data S2, and osteometric information about specimens at each locality is summarized in Data S3. J.I. Mead and J.M. Martin accrued a database of 2,400 *Bison* calcanea representing 60 localities (geological strata at geographic locations) in North America (Figure 2, Data S2). We used determinations of radiocarbon age only after 1990 for consistent accuracy of radiometric estimates (Data S2) that were calibrated using OxCal Online Tool (https://c14.arch.ox.ac.uk/) by employing the IntCal13 curve (Reimer, Bard, & Bayliss, 2013). Calibrated ages and errors are reported in Data S1; ages and errors in analyses are assumed accurate but not precise due to variability of the radiocarbon curve (Reimer et al., 2013). Specimens lacking adequate chronologies or osteometrics (<3 measures) were omitted from subsequent analyses, thus providing 1,169 samples.

**Figure 3:**
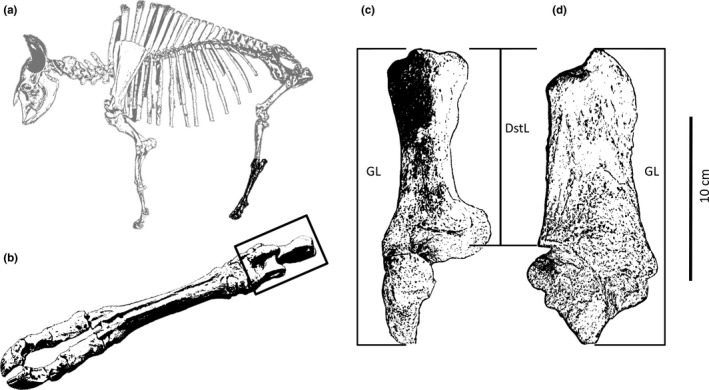
Standard metrics on a typical fossil calcaneum from a *Bison* (a) hock (b) shown in dorsal view (c) and medial view (d). Two measures for assessing body size of bison are illustrated: GL, greatest length; DstL, distal tuber length. Additional measures of the calcaneum are described by Von Den Driesch (1976) and Hill (1996)

Fossil calcanea were reported as belonging to one of three species of *Bison* (e.g., *B. bison*,* B. antiquus*, and *B. occidentalis*) in collection databases based on associated diagnostic elements with specific shape and morphological landmarks (e.g., horn cores, (Skinner & Kaisen, 1947; Balkwill & Cumbaa, 1992)). Some of the specimens were originally identified as *Bison bison antiquus*, (*nomen dubium*), which has been synonymized with *B. antiquus* (McDonald, 1981). Six standard linear measurements were taken on the calcaneum (Hill, 1996; McDonald, 1981; Miller & Brotherson, 1979; Olsen, 1960; Von Den Driesch, 1976): distal breadth of calcaneal tuber (DstBr), greatest breadth of calcaneum at the sustentaculum (GBr), distal depth of calcaneal tuber (DstDp), distal length of calcaneal tuber (DstL), greatest length of calcaneum (GL), and greatest depth of calcaneum at the sustentaculum (GDp, Figure 3). We used DstL to estimate live body mass (*M*) by the relationship of Christiansen (2002, p. 688).

(1) $$M={\left(\text{DstL}/11.49\right)}^{3}$$

We assume that global temperature is relative to the Greenland Ice Sheet Project (GISP2) ice core paleotemperature proxy data (Grootes, Stuiver, White, Johnsen, & Jouzel, 1993). Proxy data from reconstructing global paleoclimatic temperature in °C were derived from GISP2 δ^18^O values (‰; Grootes et al., 1993; Alley, 2000; Alley & Ágústsdóttir, 2005) and were related to average age of the locality. The global temperature anomaly was derived by scaling the GISP2 data to the estimated Last Glacial Maximum temperature, which was on average 6°C colder than the 20th‐century average global temperature.

We used mixed model regressions for each metric of the calcaneum to compare species as a fixed effect with *B. bison* as the base for the comparison (Stata v14.2, 2015, StataCorp, College Station, TX, USA). Similarly, mixed models were used to compare DstL with other calcaneal metrics with species as a fixed effect. The fixed effects of species, temperature, and latitude were included in the model to analyze DstL and estimates of body mass from measures of DstL (Christiansen, 2002). We used two estimates of temperature in the models: GISP2 temperatures and the relative global temperature anomaly. All mixed models included site as a random effect to account for repeated measures within each location. We used the robust “sandwich estimator” to relax assumptions of normal distribution and homogeneity of variance for the regression (Bolker et al., 2009; Rabe‐Hesketh & Skrondal, 2012). Pairwise group comparisons among predicted margins from each model were made with Bonferroni's correction (α *= 0.05*).

## 3 RESULTS

**Figure 4:**
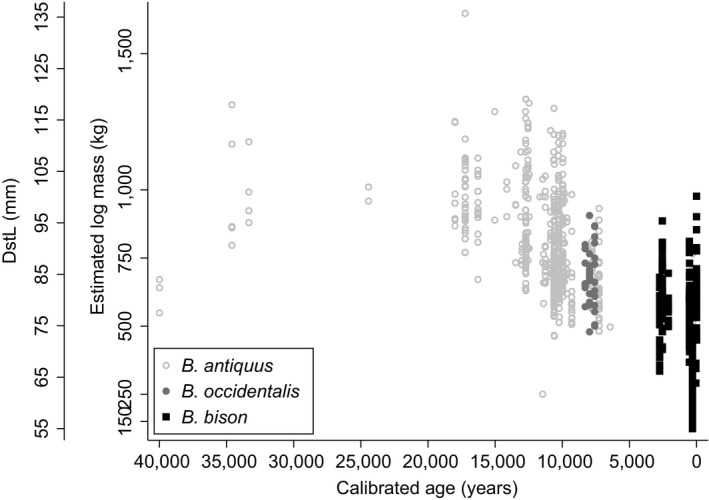
Average body size of fossil bison measured as calcaneal lengths (DstL) and body mass at 60 localities in North America from 40,000 years ago (left) to today (right)

Species significantly affected all metrics of calcaneal size (Table 1), that is, specimens from *B. antiquus* were larger than those of *B. bison*. Similarly, the intercept of the positive relationship between the depth or breadth of the calcaneum and its tuber length (DstL) was greater for *B. antiquus* than for *B. bison* (Table 2). Estimated body mass decreased over time from *B. antiquus* (802 ± 183 kg) to *B. occidentalis* (678 ± 105 kg) to modern *B. bison* (479 ± 177 kg; (Figure 4).

**Table 1:** Summary statistics [$\overset{¯}{X}$ *± SD* (*n*)] calcaneal osteometrics (mm) of *Bison*

Parameter	*Bison bison*	*B. occidentalis* a	*B. antiquus* a
GL	142.1 ± 12.2 (428) A	155.9 ± 8.7 (35) B	161.8 ± 11.3 (568) C
DstL	88.4 ± 12.0 (273) A	100.7 ± 5.3 (36) B	106.2 ± 8.0 (540) C
DstBr	36.17 ± 3.8 (164) A	38.3 ± 4.1 (35) B	41.5 ± 4.7 (569) B
DstDp	39.3 ± 3.5 (164) A	42.2 ± 3.5 (38) B	44.3 ± 4.5 (589) C
GBr	48.0 ± 4.5 (433) A	50.3 ± 4.2 (33) B	55.2 ± 5.2 (545) B
GDp	55.5 ± 4.5 (400) A	58.3 ± 4.6 (34) B	63.7 ± 5.0 (563) B

DstBr, distal tuber breadth; DstDp, distal tuber depth; DstL, distal tuber length, GBr, greatest breadth; GDp, greatest depth; GL, greatest length.

Uppercase letters indicate significant pairwise differences (*p *<* .05*) between species within each measure (row).

a Extinct.

**Table 2:** Regression relationships for estimating distal tuber length in *Bison*

Parameter	Obs.	Sites	Intercept (± *SE*)	Slope (± *SE*)
GL^a^	743	53	−6.22 ± 2.48	0.68 ± 0.01
DstBr	645	48	46.69 ± 2.06 + 3.14 ± 1.04 (*B.a*.) + 2.37 ± 1.18 (*B.o*.)	1.34 ± 0.05
DstDp	662	47	40.38 ± 2.78 + 4.53 ± 1.52 (*B.a*.) + 2.45 ± 1.45 (*B.o*.)	1.36 ± 0.07
GBr	714	51	40.13 ± 4.57 + 6.00 ± 1.93 (*B.a*.) + 5.99 ± 2.00 (*B.o*.)	1.08 ± 0.07
GDp	723	52	29.49 ± 4.88 + 5.77 ± 2.29 (*B.a*.) + 6.30 ± 2.35 (*B.o*.)	1.11 ± 0.07

B.a., Bison antiquus; B.o., B. occidentalis.

(DstL) from other measures of the calcaneum (GL, DstBr, DstDp, GBr, GDp) in *Bison* using mixed models with site as a random effect and *B. bison* as the comparison base for species.

a No linear effect of species on GL (*p *<* .05*).

**Figure 5:**
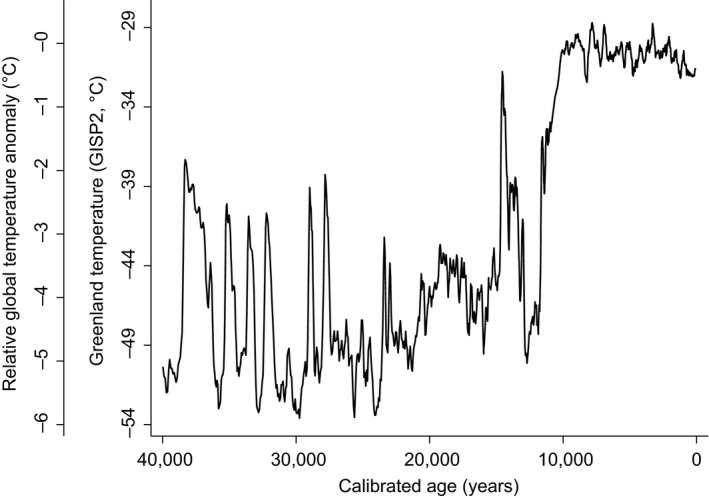
Sequence of Greenland mean annual temperature (°C derived from GISP2 δ^18^O values (Alley & Ágústsdóttir, 2005)) and relative global temperature anomaly derived from modern Greenland temperatures (μ29.9°C mean annual temperature) from 40,000 years ago (left) to today (right)

The greatest proportion of specimens (50%) were those of *B. antiquus* and *B. occidentalis* that were dated between 7,000 and 13,000 years ago, whereas 38% of the specimens were those of *B. bison* from 3,000 years ago to present. Average annual temperatures varied over 25°C on the scale of Greenland temperature over the last 40,000 years, which was equivalent to a span of 6°C on the relative global scale (Figure 5).

The largest proportion of *Bison* specimens were associated with two large fluctuations from 15,000 years ago to present that included warming in the Bølling–Allerød period (15,000 years to 13,000 years ago), cooling in the Younger Dryas (13,000 to 12,000 years ago), and warming through the Holocene period to present with small undulations in temperature, such as the Medieval Climatic Anomaly (approximately 1,000 to 700 years ago) and Little Ice Age (approximately 700 to 150 years ago; Figure 5).

**Figure 6:**
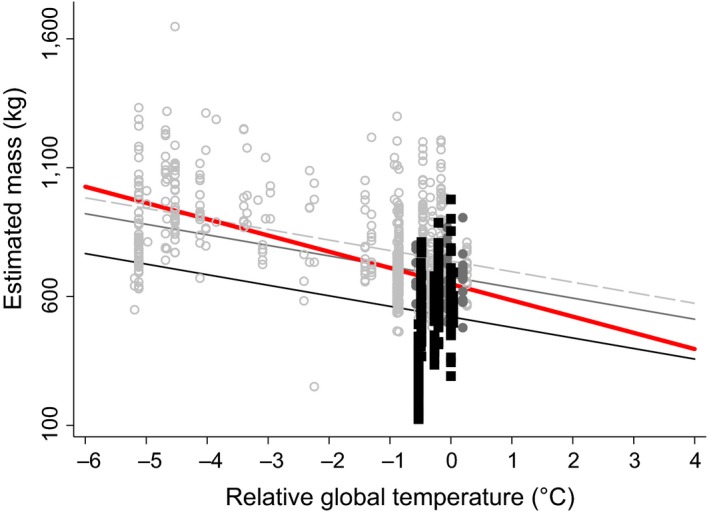
Relationship between estimated body mass (kg; ± *SE*) and the linear effect of relative global temperature (°C derived from GISP2 δ^18^O values) from the mixed model regression with fixed effects of temperature and the random effect of site. Regression line (*y* = −40.9 kg/°C ± 10) with lines for specific regressions (intercepts for *B. bison* (black): 520.9 *±* 36.1; *B. occidentalis* (dark gray): 675.6 *±* 36.2; *B. antiquus* (light gray): 737.3 *±* 44.7; *p *<* *.001, *n *=* *849, *N *=* *53). Regression line for the small‐sized *Bison* clade (red line) is −63 kg/°C (± 10; *z *=* *−6.11 *p *<* *.001) with an intercept at 648 ± 26 kg

Calcaneal distal tuber length (DstL) was negatively related to Greenland temperature (slope: −0.45 mm/°C ± 0.11; *z *= −3.95 *p *<* *.001) with intercepts at 78 ± 4 mm for *B. bison*, 90 ± 3 mm for *B. antiquus,* and 87 ± 2 mm for *B. occidentalis*. The relationship between calcaneal distal tuber length (DstL) and relative global temperature was −1.77 mm/°C ± 0.45 (*z *=* *−3.95, *p *<* *.001) with intercepts at 92 ± 2 mm for *B. bison*, 103 ± 3 mm for *B. antiquus,* and 101 ± 2 mm for *B. occidentalis*. Consequently, the slope of estimated body mass with global temperature was also negative at −41 kg/°C (± 10; *z *=* *−4.10, *p *<* *.001) with intercepts at 521 ± 36 kg for *B. bison*, 737 ± 45 kg for *B. antiquus,* and 676 ± 36 kg for *B. occidentalis* (Figure 6). This relationship predicts that *B. bison* will decrease by 164 ± 40 kg to 357 ± 54 kg if global temperature rises from 0°C to +4°C (Figure 6).

## 4 DISCUSSION

Our data supported our hypothesis that global climate change drives body size of *Bison* spp., that is, as temperatures warmed, *Bison* became smaller. Generally, described as Bergmann’s Rule (Bergmann, 1847), endotherms increase in body size with increasing latitude (Huston & Wolverton, 2011). It is likely that negative correlation between temperature and latitude is driving Bergmann's rule (i.e., body size) because even though we found that bison are larger at cooler temperatures, we were unable to correlate a significant effect of latitude over the geologic record (*p *>* *.94). The negative relationship between body mass and global temperature may reflect underlying relationships between body size and net primary production as well as heat loads (Speakman & Król, 2010; Huston & Wolverton, 2011; Figure 1).

Paleontologists have long used skeletal elements from extant animals to reconstruct body mass and body shape of fossils (Christiansen, 2002; Damuth & MacFadden, 1990; Gingerich, 1990). Data from some bones indicate body size more accurately than others. Indices of body size in mammals, including *Bison,* are best indicated by bones of the hind foot (elements of the ankle, calcaneum, and astragalus), and front foot (elements of the wrist, scaphoid, and magnum), along with the toes (podial digits and distal and proximal phalanges; (Damuth & MacFadden, 1990)). The bulk of the foot bones precisely reflects body mass because they bear the weight of the animal, whereas the shape of the bones reflects the functional anatomy for locomotion through the attachment of tendons and muscle (Scott, 1990). Longer bones of limbs (femora and humeri) are also good proxies for reconstructing body size. Unfortunately, long bones in the fossil record are typically broken, whereas the calcanea, astragali, and phalanges are commonly well preserved, likely because these dense elements resist degradation. Consequently, podial elements are well studied within Bovinae, which includes cattle (*Bos taurus*, (Lawrence, 1951; Olsen, 1960; Balkwill & Cumbaa, 1992)), and Antilopinae, mountain goats (*Oreamnos* sp., (Carpenter, 2003)), bighorn sheep (*Ovis* sp., (Todd & Rapson, 1988; Rothschild & Martin, 2003), among others). However, it is difficult to distinguish taxa using podial elements. *Bison* and *Bos* can be resolved from traits of podial elements by the methods of Balkwill and Cumbaa (1992) but we cannot resolve *Bison* species based upon podial elements alone. Species designations in our dataset originated from whole collections of associated podial and cranial material that may not distinguish mixes of species at each location. For example, American Falls Reservoir in Idaho contains at least four co‐existing species of *Bison* (Pinsof, 1991). If we ignore species designations and analyze our data at the clade level, the slope of podial size with increasing temperatures becomes steeper; −63 kg/°C (±10; *z *=* *−6.11 *p *<* *.001) with an intercept at 648 ± 26 kg for *Bison* spp., as compared to the −41 kg/°C for *Bison bison* (Figure 6). This slope may change regionally with latitudinal differences in body size of extant *Bison*.

*Bison* crania exhibit plastic morphology, likely due to a combination of environmental and sexual selection, whereas postcranial elements—podial elements specifically—exhibit a more conservative and accurate reflection of body size due to functional anatomy of the appendicular skeleton (Clifford, 2009, 2010). Historically, it has been difficult to identify *Bison* fossil species (*Bison bison*,* B. occidentalis*, and *B. antiquus*) based on skeletal remains without skulls, especially those without horn cores (McDonald, 1981; Skinner & Kaisen, 1947). This issue continues today (Grayson, 2006; Lyman, 2004; McDonald & Lammers, 2002), with the exceptions of *B. latifrons* (Giant bison (Hopkins, 1951; Schultz & Hillerud, 1977; Pinsof, 1991)) and *B. priscus* (Steppe bison; (Gee, 1993; Zazula, MacKay, & Andrews, 2009; Boeskorov et al., 2014)), which are distinct because of their massive size. Many of the above authors rely on cranial elements alone to specifically classify *Bison*, but recent studies suggest that the diagnostic *Bison* cranial characters vary widely (Krasinska, 1988) and do not reflect conservative morphological variability in the skeleton. Cranial elements of *Bison* are now thought too variable to rely on for taxonomic classification (Prothero & Foss, 2007). Widga (2013) attempted to synthesize a large dataset of bison horn‐core metrics and illustrates the noise inherent in these samples (Hill, Hawley, Widga, Monahan, & Wanamaker, 2014; Wilson, 1974).

Some researchers suggest that the past several millennia of anthropogenic selection by Paleoindians, conservationists, and producers may have directly and indirectly selected traits that scale to body size (i.e., large heart girths, large heads, straight vertebral column; (Todd, 1983; Grayson, 2000, 2001)). Undoubtedly, early arrivals of modern humans were having impacts on the available bison through hunting some 14,000 years ago (Barnosky et al., 2014; Grayson, 2000); however, these effects were limited by small human populations dispersed over a large continent and were therefore local impacts (Hawley, Hill, & Widga, 2013; Hill et al., 2008, 2014). Others have acknowledged that any selection has not made significant changes in morphology (Hawley et al., 2013; Hill et al., 2008, 2014). Climate is the most parsimonious explanation for shaping *Bison* morphology (Hill et al., 2008; Lewis et al., 2007; Shapiro et al., 2004). Changes in body size of *Bison* could be a result of migration or disease but those effects are geographically local and not likely to persist over the long time scale of the fossil record (Hamel et al., 2016). Wilson, Hills, and Shapiro (2008) postulate the decrease in body size of *Bison* is a consequence of dispersal theory, that is, expansion of range, over the last 80,000 years (Wilson, 1996). A more cogent argument explaining decrease in body size is the rapidly warming global climate, characterizing the termination of the Younger Dryas period.

This study demonstrates a strong inverse correlation between increasing global temperatures and body size of bison over the last 40,000 years. We hypothesize that increasing temperature alters both metabolic demands and available resources (Figure 1).

The IPCC Working Group 1 (2014) predicts 4°C rise in global temperatures by year 2100. While the absolute increase in 4°C is not unprecedented in the evolutionary history of *Bison*, the rate of temperature change is 30 times faster than the Bølling–Allerød period, the transition from the Last Glacial Maximum to Holocene climate conditions. The Last Glacial Maximum corresponds with a global temperature 6°C cooler than the 20th century, when *Bison* mass was 910 kg. If global temperature warms to +4°C as predicted for the 21st century, *Bison* body mass will likely decline from 665 kg to 357 kg (Figure 6), if body size declines at the long‐term average. The greatest decline in body size of *Bison* apparently occurred between 12,500 and 9,250 years ago, when mass declined by 26% (906 kg to 670 kg) in approximately 3,000 years. If generation time of *Bison* is 3–10 years (Evans et al., 2012; Gingerich, 1993), the change in body size occurred in 325–1,080 generations producing an average rate of change of 0.2–0.7 kg per generation. It is unclear whether *Bison* can adapt body size to a 4°C warming within 10 generations by year 2100.

*Bison* today express a 30% body mass gradient from north to south, that is, *Bison* in Saskatchewan (52°N) are at least 30% larger than those in Texas (30°N (Craine, 2013, p. 3)). This body size gradient is likely associated with latitudinal variation in timing of reproduction and parturition as well as windows for growth (Barboza et al., 2009). Quantifying and comparing physiological thresholds and mechanisms driving body size change are imperative for managing *Bison* and other large herbivores (Figure 1). Conservation goals among latitudinally disparate *Bison* herds in North America should consider that resident *Bison* will likely grow smaller and more slowly in the south than in the north, which will impact management strategies at both regional and continental scales.

## CONFLICT OF INTEREST

None declared.

## AUTHOR CONTRIBUTIONS

JMM conceived and developed the study. JMM and JIM acquired the data. JMM, JIM, and PSB interpreted and analyzed the data. JMM, JIM, and PSB drafted, revised, and approved the final manuscript.
